# Delayed initiation of adjuvant chemotherapy in older women with breast cancer

**DOI:** 10.1002/cam4.3363

**Published:** 2020-08-07

**Authors:** Demetria Smith‐Graziani, Xiudong Lei, Sharon H. Giordano, Hui Zhao, Meghan Karuturi, Mariana Chavez‐MacGregor

**Affiliations:** ^1^ Division of Cancer Medicine The University of Texas MD Anderson Cancer Center Houston TX USA; ^2^ Department of Health Services Research The University of Texas MD Anderson Cancer Center Houston TX USA; ^3^ Department of Breast Medical Oncology The University of Texas MD Anderson Cancer Center Houston TX USA

**Keywords:** breast, chemotherapy, delay, disparities, elderly

## Abstract

**Background:**

Adjuvant chemotherapy benefits early‐stage breast cancer (BC) patients. Older women receive guideline‐adherent treatment less frequently and experience treatment delays more frequently. We evaluated factors associated with delaying adjuvant chemotherapy and the delays’ survival impact in a large population–based cohort of elderly BC patients.

**Methods:**

Patients age >66 years diagnosed 2001‐2015 with localized or regional BC were identified in the SEER‐Medicare and Texas Cancer Registry‐Medicare databases. Time from surgery to chemotherapy (TTC) was categorized into four groups: 0‐30, 31‐60, 61‐90, and >90 days. We identified predictors of delays, estimated overall (OS) and BC‐specific (BCSS) survival, and determined the association between TTC and outcome adjusting for other variables.

**Results:**

Among 28,968 women (median age 71 years), median TTC was 43 days. 10.7% of patients experienced TTC >90 days. Older age, Black or Hispanic race/ethnicity, unmarried status, more comorbidities, hormone receptor‐positivity, mastectomy, Oncotype DX testing, and full state buy‐in were associated with increased risk of delay. Five‐year OS estimates by TTC group were 0.82, 0.81, 0.80, and 0.74, respectively (p<.001). BCSS demonstrated a similar trend (p<.001). Chemotherapy delay was associated with worse OS (HR=1.33, 95%CI 1.25‐1.40) and BCSS (HR=1.39, 95%CI 1.27‐1.53). In subgroup analysis, delayed chemotherapy was associated with worse OS and BCSS among patients with hormone receptor–positive (HR=1.56, 95%CI 0.97‐2.51), HER2‐positive (HR=1.99, 95%CI 1.04‐3.79), and triple‐negative (HR=2.15, 95%CI 1.38‐3.36) tumors.

**Conclusion:**

Chemotherapy delays are associated with worse survival in older BC patients. Providers should avoid delays and initiate chemotherapy ≤90 days after surgery regardless of patients’ BC subtype or age.

## Graphical abstract

We evaluated the factors associated with delays in the initiating adjuvant chemotherapy and the survival impact of delays in a large population‐based cohort of elderly breast cancer patients. We found chemotherapy delays beyond 90 days after surgery negatively affected survival, and delays were associated with clinical and socioeconomic factors.

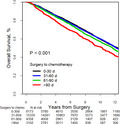

## 1 INTRODUCTION

Adjuvant chemotherapy is known to decrease both mortality and risk of recurrence in patients with early‐stage breast cancer (BC).^1^ Despite the benefits of adjuvant chemotherapy, there is concern that delays in initiating adjuvant chemotherapy can negatively affect survival. Prior studies show that delaying adjuvant chemotherapy beyond 60^2^, ^3^ or 90^4^ days leads to worse outcomes. In a large analysis using the California Cancer Registry (CCR), our group described that delays in chemotherapy administration affect survival outcomes with a particular detriment among patients with stage III disease or HER2‐positive or triple negative BC (TNBC).^4^ Data among BC patients enrolled in Medicaid is consistent with such observations, suggesting that a time to chemotherapy (TTC) >60 days after surgery is associated with decreased survival.^5^ There are, however, some retrospective studies that have not demonstrated significant harm associated with delays in treatment.^6^, ^7^ Given conflicting data, additional studies are warranted to investigate potential associations between timing of chemotherapy and clinical outcomes.

Although adjuvant chemotherapy has proven benefit in older women, these patients are not always treated according to established guidelines.^8^, ^9^ Among a cohort of 1800 postmenopausal BC patients, older patients were less likely to be treated according to consensus statements.^10^ A study of women ≥65 years with stages II‐III BC found that only 43% of patients received guideline‐recommended adjuvant chemotherapy and older patients were less likely to receive treatment within 4 months of diagnosis.^11^ While fewer older women present with lymph node involvement, a retrospective cohort study found no evidence that older BC patients had more indolent disease.^12^ Therefore, it is of foremost importance to evaluate the impact of treatment delays among elderly BC patients and identify risk factors for such delays. Prior studies have identified socioeconomic status (SES),^11^ race,^4^, ^6^, ^13^ comorbidities,^7^, ^11^ tumor biomarkers,^5^ distance to hospital, and type of insurance as factors associated with longer TTC.^3^ We evaluate the survival impact of delays in adjuvant chemotherapy initiation among older BC patients and identify factors associated with such delays.

## 2 METHODS

We used the National Cancer Institute's Surveillance, Epidemiology, and End Results (SEER)‐Medicare and the Texas Cancer Registry (TCR)‐Medicare linked databases. Female patients diagnosed with localized or regional BC from 2001 to 2015 and age ≥66 years at the time of diagnosis were identified. We included patients for whom BC was their first primary cancer and excluded those with a second histology‐confirmed cancer within 12 months of their BC diagnosis. To ensure data completeness, we included only patients who had continuous Medicare Part A & B coverage without health maintenance organization enrollment 12 months before and 12 months after diagnosis. All included patients had a mastectomy or lumpectomy and received adjuvant chemotherapy within 9 months of primary breast surgery. Patients who received neoadjuvant chemotherapy were excluded. The final cohort included 28 968 patients (Appendix Table S1).

### 2.1 Outcome and variables of interest

We defined overall survival (OS) and breast cancer‐specific survival (BCSS) as the time from date of diagnosis to date of death (for OS) or date of death due to BC (for BCSS), or date of last follow‐up. Patients who did not die or did not die due to breast cancer‐specific cause (for BCSS) were censored at the last follow‐up time. The last follow‐up date for OS was 31 December 2017 using the date of death documented by Medicare. However, since the cause of death was obtained from SEER, the last follow‐up date for BCSS was 31 December 2015.

Clinical and demographic variables including region, year and age of diagnosis, race, marital status, stage, state buy‐in (as a proxy for poverty), and level of education were extracted from the SEER or TCR patient enrollment file. We estimated the percentage of non‐high school education based on 2000 census tract. We used physician, inpatient, and outpatient claims in the 12 months before diagnosis to calculate Charlson Comorbidity Index score.^14^ We used Medicare claims to identify the type of surgery, Oncotype DX testing, and radiation status and Emergency room visits/hospitalizations and surgical complications (bleeding, cellulitis, abscess, wound dehiscence, seroma, infection, and inflammatory reaction due to other prosthetic device/implant/graft) between the day of surgery and the day chemotherapy was initiated (Appendix Table S2). We identified chemotherapy use using procedure codes and recorded the date chemotherapy was initiated. The time between final breast surgery and first chemotherapy dose was calculated; we grouped patients according to time from surgery to chemotherapy in 4 categories: 0‐30, 31‐60, 61‐90, and >90 days. Delayed chemotherapy initiation was defined as TTC > 90 days.

### 2.2 Statistical methods

Descriptive statistics were used; we used the Chi‐square test to compare categorical variables. Multivariable logistic regression model was used to identify predictors of delayed chemotherapy. Variables in the model included: year of diagnosis, age at diagnosis, race, marital status, Charlson comorbidity index, tumor size, lymph node status, grade, hormone receptor status, type of surgery, Oncotype DX test, administration of radiation therapy prior to chemotherapy, state buy‐in, residence, and education. In addition, we included emergency room visits/hospitalization/surgical complication between date of surgery and first chemotherapy claim as an additional variable.^15^, ^16^ Results are presented in odds ratios (OR) and 95% confidence intervals (CI).

We calculated and plotted the five‐year OS and BCSS estimates using the Kaplan‐Meier product‐limit method and Cox proportional hazards models were used to determine the association of delayed chemotherapy with OS and BCSS, adjusting for the previously mentioned covariates. We included emergency room visits/hospitalization/surgical complication as a time‐varying covariate in the model. We expressed results in hazard ratios (HR) and 95% CIs. We adjusted for potential selection bias by the inverse probability treatment weighted (IPTW) method. To obtain the probability of patients falling in each of the three TTC groups, we implemented three separate logistic regression models using TTC 0‐30 days as the reference group. We obtained propensity scores (PS) for each model and defined the IPTW weights as PS/(1‐PS) for patients in each of the three groups (31‐60, or 61‐90, or >90 days), assigning patients initiating chemotherapy in 0‐30 days a weight of 1.^17^ We verified the post adjustment balance using the standardized difference, with a standardized difference <10% indicating good balance. Sensitivity analysis further categorized delayed chemotherapy into 91‐120, 121‐180, >180 days.

We evaluated the association between TTC and survival outcomes within subgroups stratified by hormone receptor status and stage. In addition, we performed a subgroup analysis among patients diagnosed from 2010 to 2015 in whom information on HER2 status was available. We evaluated the association of chemotherapy delays and survival outcomes stratified by BC subtype. We considered *P* < .05 statistically significant. We used SAS version 9.4 (SAS Institute), and R version 3.5.1. We received Institutional Review Board exception based on the code of regulations.

## 3 RESULTS

**FIGURE 1:**
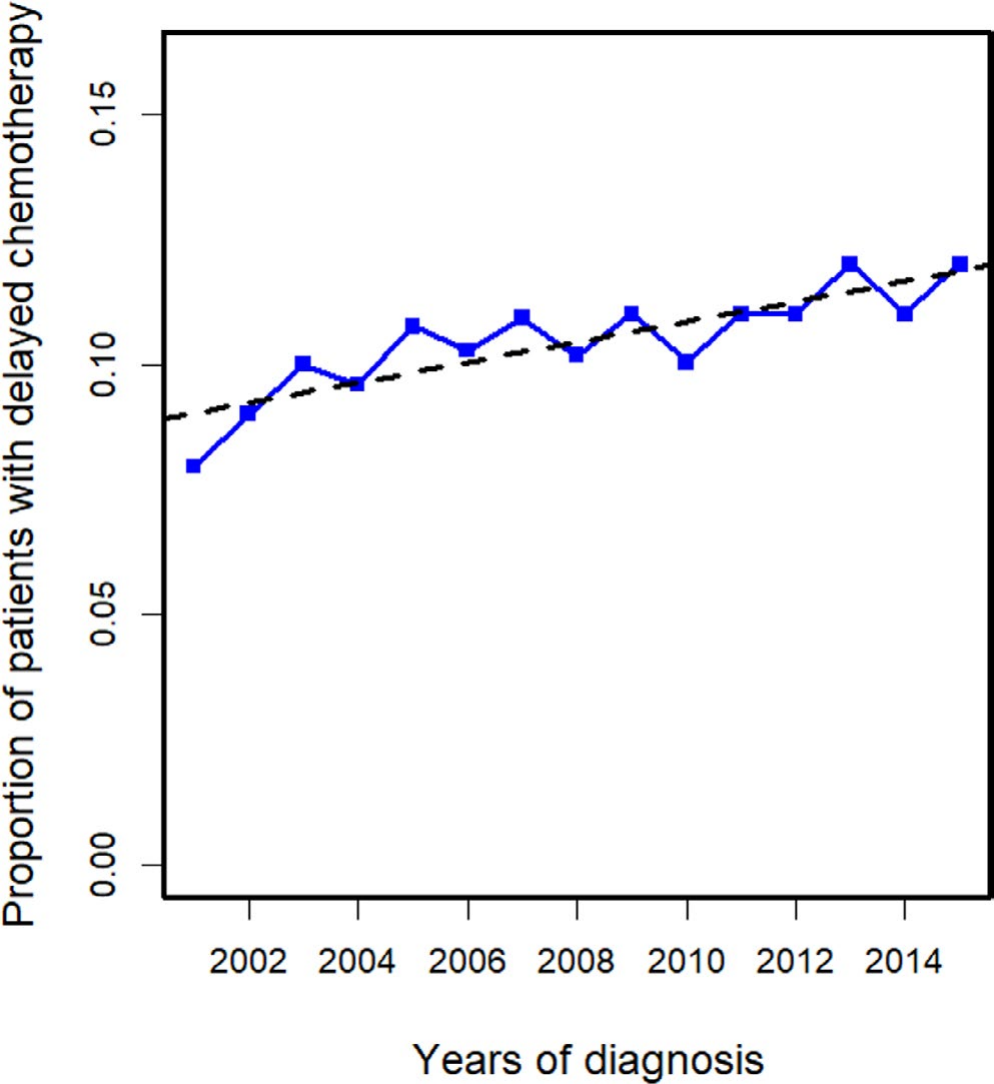
Plot of percentage of patients who had delayed chemotherapy (TTC > 90 d) according to year of diagnosis

In total, 28 968 women were included. Table 1 lists the baseline characteristics of the cohort by TTC. The median age of the cohort was 71 years (range 66‐96 years). The median TTC was 43 days (range 0‐273 days). This interval increased from 38 days in 2001 to 42 days in 2005, and stabilized around 48 days in 2015 (Figure 1). The percentage of patients who experienced a delay in TTC increased from 8% in 2001 to 12.3% in 2015 (*P* < .001); 3102 (10.7%) patients overall delayed chemotherapy. Before weighting, all variables across the different TTC groups were significantly different (*P* < .001). After IPTW adjustment, greater balances were achieved, with standardized difference <10%.

**TABLE 1:** Unadjusted (unweighted) patient and clinical characteristics by time to chemotherapy (TTC) (N = 28 968)

	TTC			
	0‐30 d (%) (N = 6699) N (%)	31‐60 d (%) (N = 14 236) N (%)	61‐90 d (%) (N = 3974) N (%)	>90 d (%)(N = 3033) N (%)
	N = 6173	N = 15 335	N = 4358	N = 3102
Year of diagnosis				
2001	570 (31.3)	894 (49.1)	212 (11.6)	145 (8)
2002	557 (30.8)	866 (47.9)	210 (11.6)	176 (9.7)
2003	546 (32.5)	757 (45)	202 (12)	177 (10.5)
2004	700 (30.6)	1117 (48.9)	248 (10.9)	219 (9.6)
2005	525 (24.6)	1088 (51.1)	289 (13.6)	229 (10.7)
2006	396 (18.8)	1157 (55)	335 (15.9)	216 (10.3)
2007	360 (17.1)	1189 (56.4)	328 (15.6)	230 (10.9)
2008	405 (19.4)	1125 (54)	341 (16.4)	212 (10.2)
2009	332 (16.2)	1135 (55.5)	345 (16.9)	234 (11.4)
2010	307 (15.9)	1091 (56.4)	341 (17.6)	194 (10)
2011	336 (17.3)	1066 (54.8)	320 (16.5)	223 (11.5)
2012	348 (17.4)	1110 (55.4)	316 (15.8)	231 (11.5)
2013	321 (16.3)	1070 (54.3)	323 (16.4)	255 (13)
2014	232 (15.4)	836 (55.4)	269 (17.8)	171 (11.3)
2015	238 (15.4)	834 (54.1)	279 (18.1)	190 (12.3)
Age at surgery (y)				
66‐70	2865 (21.9)	7230 (55.2)	1936 (14.8)	1070 (8.2)
71‐75	1846 (21)	4741 (54)	1354 (15.4)	831 (9.5)
76‐80	1001 (20.7)	2422 (50.2)	732 (15.2)	670 (13.9)
>80	461 (20.3)	942 (41.5)	336 (14.8)	531 (23.4)
Race/ethnicity				
Non‐Hispanic white	5060 (22)	12 222 (53.2)	3313 (14.4)	2396 (10.4)
Non‐Hispanic black	466 (17.6)	1380 (52.2)	471 (17.8)	325 (12.3)
Hispanic	429 (20)	1094 (51.1)	357 (16.7)	260 (12.1)
Other	218 (18.2)	639 (53.5)	217 (18.2)	121 (10.1)
Marital status				
Married	3265 (22.8)	7681 (53.7)	2039 (14.3)	1314 (9.2)
Single	2394 (20)	6188 (51.7)	1892 (15.8)	1491 (12.5)
Unknown	514 (19)	1466 (54.2)	427 (15.8)	297 (11)
Charlson comorbidity score				
0	3979 (22.4)	9514 (53.7)	2509 (14.2)	1722 (9.7)
1	1380 (20.5)	3485 (51.8)	1078 (16)	788 (11.7)
2+	625 (16.7)	1927 (51.6)	667 (17.8)	519 (13.9)
Unknown	189 (24.4)	409 (52.8)	104 (13.4)	73 (9.4)
Tumor size				
0‐20 mm	2815 (20.8)	6991 (51.6)	2095 (15.4)	1660 (12.2)
21‐50 mm	2692 (21.5)	6869 (54.7)	1859 (14.8)	1128 (9)
>50 mm	512 (23)	1157 (52.1)	321 (14.4)	232 (10.4)
Unknown	154 (24.2)	318 (49.9)	83 (13)	82 (12.9)
Lymph node status				
Negative	2265 (18.9)	6113 (51)	1919 (16)	1689 (14.1)
Positive	3159 (23.3)	7431 (54.9)	1933 (14.3)	1013 (7.5)
Unknown	749 (21.7)	1791 (52)	506 (14.7)	400 (11.6)
ER/PR status				
Negative	1679 (23.3)	4042 (56.2)	963 (13.4)	511 (7.1)
Positive	3548 (20.2)	9136 (52)	2785 (15.8)	2114 (12)
Unknown	946 (22.6)	2157 (51.5)	610 (14.6)	477 (11.4)
Surgery				
Lumpectomy	3085 (21)	7529 (51.4)	2275 (15.5)	1767 (12.1)
Mastectomy no reconstruction	2936 (21.8)	7344 (54.4)	1947 (14.4)	1266 (9.4)
Mastectomy with reconstruction	152 (18.6)	462 (56.4)	136 (16.6)	69 (8.4)
Emergency room/ hospitalization/complication				
No	23 386 (80.7)	5527 (89.5)	12 790 (83.4)	3138 (72)
Yes	5582 (19.3)	646 (10.5)	2545 (16.6)	1220 (28)
Oncotype DX test				
No	6068 (22.8)	14 101 (52.9)	3743 (14.1)	2719 (10.2)
Yes	105 (4.5)	1234 (52.8)	615 (26.3)	383 (16.4)
Radiation before chemo				
No	6121 (22.8)	14 866 (55.3)	4006 (14.9)	1892 (7)
Yes	52 (2.5)	469 (22.5)	352 (16.9)	1210 (58.1)
State buy‐in				
None/partial	5518 (21.7)	13 532 (53.1)	3763 (14.8)	2651 (10.4)
Full	655 (18.7)	1803 (51.5)	595 (17)	451 (12.9)
Residence area				
Big metro	3037 (20.2)	7861 (52.3)	2371 (15.8)	1771 (11.8)
Metro	1991 (22.2)	4775 (53.3)	1296 (14.5)	898 (10)
Urban	422 (23.8)	949 (53.5)	243 (13.7)	161 (9.1)
Small urban	585 (22.1)	1461 (55.2)	373 (14.1)	226 (8.5)
Rural	138 (25.2)	289 (52.7)	75 (13.7)	46 (8.4)
Percent non‐high school graduates				
1st Quartile	1412 (20.9)	3591 (53.1)	1014 (15)	741 (11)
2nd Quartile	1412 (20.9)	3648 (54)	1012 (15)	687 (10.2)
3rd Quartile	1373 (20.4)	3545 (52.6)	1045 (15.5)	775 (11.5)
4th Quartile (least educated)	1396 (20.8)	3568 (53.2)	1042 (15.5)	701 (10.5)
Unknown	580 (28.9)	983 (49)	245 (12.2)	198 (9.9)

Note

All comparisons significant (*P* < .001).

Abbreviations: ER, estrogen receptor; PR, progesterone receptor; TTC, time to chemotherapy.

### 3.1 Factors associated with delayed chemotherapy

Factors statistically associated with delayed TTC included older age (OR = 2.94, 95% CI 2.56‐3.38), Black (OR = 1.35, 95% CI 1.16‐1.57) or Hispanic race (OR = 1.26, 95% CI 1.07‐1.49), higher comorbidity score (OR = 1.21, 95% CI 1.06‐1.37), positive hormone receptor status (OR = 1.55, 95% CI 1.37‐1.74), mastectomy with immediate reconstruction (OR = 1.36, 95% CI 1.04‐1.79), having Oncotype DX testing or radiation before chemotherapy (OR = 1.36, 95% CI 1.17‐1.58 and OR = 17.38, 95% CI 15.49‐19.50), and full state buy‐in (OR = 1.14, 95% CI 1.00‐1.31). Patients experiencing surgical complications or having emergency room visits/hospitalizations after surgery had and increased risk TTC delay (OR = 3.31, 95% CI 3.02‐3.63). The full multivariable model is shown in Table 2.

**TABLE 2:** Logistic regression model evaluating predictors of time to chemotherapy delay (TTC ≥ 90 d)

	Odds ratio	95% CI	*P*
Age at surgery (y)			
66 to 70	1		
71 to 75	1.17	1.05 to 1.3	.004
76 to 80	1.63	1.45 to 1.84	<.001
>80	2.94	2.56 to 3.38	<.001
Race/ethnicity			
Non‐Hispanic white	1		
Non‐Hispanic black	1.35	1.16 to 1.57	<.001
Hispanic	1.26	1.07 to 1.49	.006
Other	1.07	0.86 to 1.33	.56
Marital status			
Married	1		
Single/divorced/widow	1.19	1.09 to 1.31	<.001
Charlson comorbidity score			
0	1		
1	1.15	1.03 to 1.27	.01
2+	1.21	1.06 to 1.37	.003
Tumor size			
0 to 20 mm	1		
21 to 50 mm	0.93	0.84 to 1.03	.15
>50 mm	0.93	0.78 to 1.11	.44
Lymph node status			
Negative	1		
Positive	0.6	0.54 to 0.66	<.001
Tumor grade			
I	1		
II	0.77	0.67 to 0.88	<.001
III to IV	0.62	0.54 to 0.72	<.001
ER/PR status			
Negative	1		
Positive	1.55	1.37 to 1.74	<.001
Surgery			
Lumpectomy	1		
Mastectomy no reconstruction	1.33	1.2 to 1.48	<.001
Mastectomy with reconstruction	1.36	1.04 to 1.79	.024
Emergency room/hospitalization/complications			
No	1		
Yes	3.31	3.02 to 3.63	<.001
Oncotype DX test			
No	1		
Yes	1.36	1.17 to 1.58	<.001
Radiation before chemo			
No	1		
Yes	17.38	15.49 to 19.50	<.001
State buy‐in			
None/partial	1		
Full	1.14	1.00 to 1.31	.06
Residence area			
Big metro	1		
Metro	0.9	0.81 to 0.99	.03
Urban	0.79	0.65 to 0.96	.018
Small urban	0.74	0.63 to 0.88	<.001
Rural	0.82	0.58 to 1.16	.26
Percent non to high school graduates			
1st Quartile	1		
2nd Quartile	0.98	0.87 to 1.12	.81
3rd Quartile	1.15	1.01 to 1.31	.034
4th Quartile (least educated)	1.05	0.92 to 1.21	.46

Note

Variables in the model also included year of diagnosis.

Abbreviations: CI, confidence interval; ER, estrogen receptor; PR, progesterone receptor.

### 3.2 OS and BCSS

**FIGURE 2:**
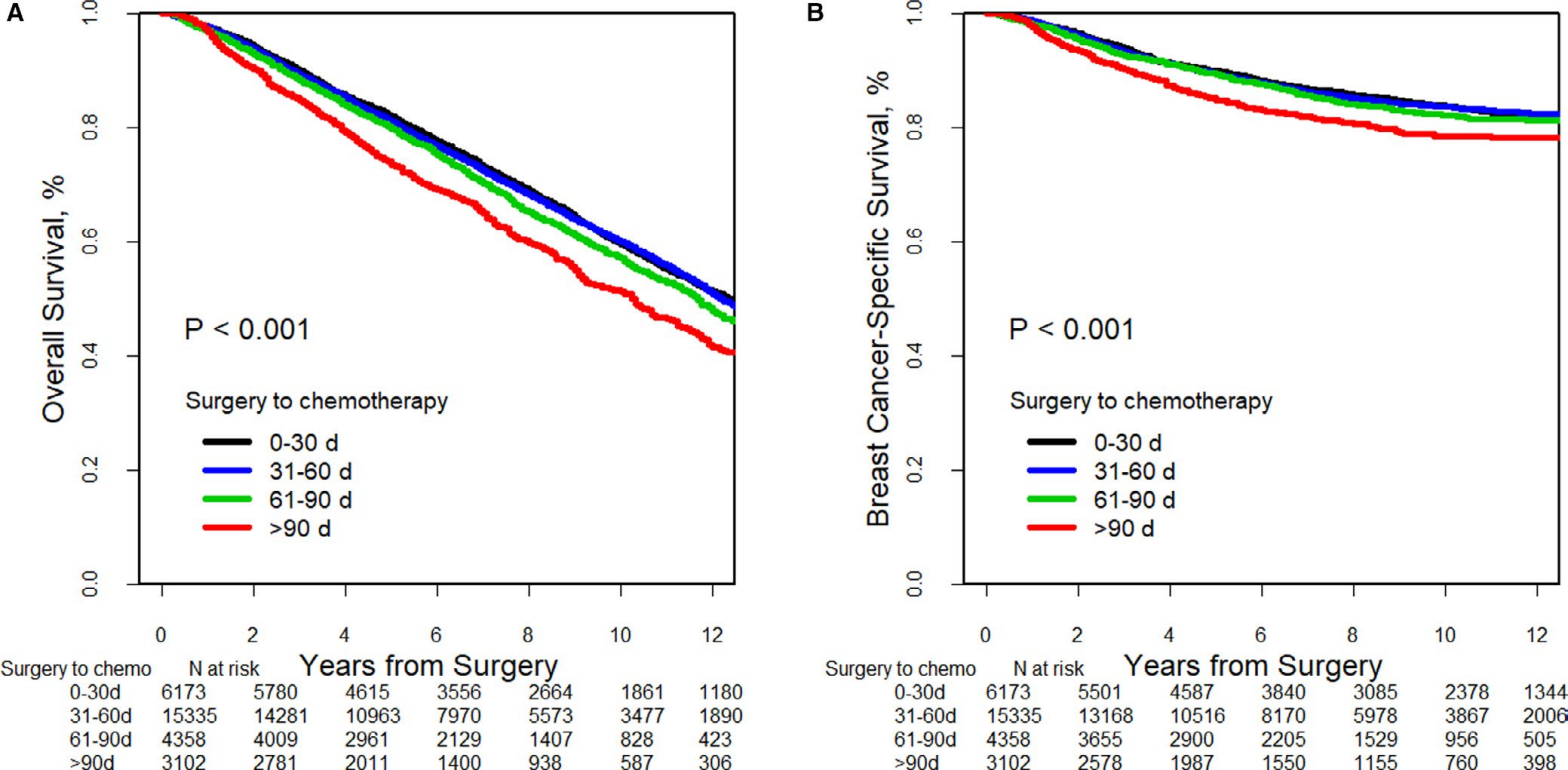
Adjusted overall survival and breast cancer‐specific survival according to time to chemotherapy. A, Overall survival. B, Breast cancer‐specific survival

Median follow‐up time was 6.2 years (range 0.1‐17 years) for OS and 5.3 years (range 0.1‐15 years) for BCSS. Overall, there were 10 425 total deaths, and 3582 of them were BC‐related. The weighted 5‐year OS and BCSS estimates for the entire cohort were 79% and 88%, respectively. On univariate log‐rank analysis, the weighted 5‐year OS estimates were 82%, 81%, 80%, and 74% for TTC 0‐30, 31‐60, 61‐90, and >90 days, respectively (*P* < .001) (Figure 2A). The 5‐year BCSS estimates were 89%, 89%, 89%, and 84% for TTC in the same time intervals (*P* < .001) (Figure 2B). To further explore the impact of delays beyond 90 days we compared survival outcomes between patients who received chemotherapy ≤90 days, to those that received it 91‐120 days (n = 1300), 121‐180 (n = 991), and > 180 days (n = 811) after surgery, 5‐year OS estimates were 82%, 78%, 72%, and 68%, respectively (*P* < .001). 5‐year BCSS estimates were 89%, 88%, 86%, and 81%, respectively (*P* < .001).

In multivariable Cox proportional hazards model analysis, after weighting and covariate adjustment (Table 3), patients with delayed TTC had an increased risk of death compared to those with TTC 0‐30 days (HR = 1.25; 95% CI 1.18‐1.33) and increased risk of BC death (HR = 1.33; 95% CI 1.20‐1.46). We performed an exploratory analysis to determine the impact of delays beyond 90 days and observed that after adjusting for important covariates, greater delays were associated with even worse outcomes (Appendix Table S3). Similar OS estimates were observed in subgroup analyses among patients with hormone receptor positive (HR = 1.44; 95% CI 1.34‐1.56) and hormone receptor negative (HR = 1.17; 95% CI 1.04‐1.30), as well as patients with localized (HR = 1.19; 95% CI 1.07‐1.32) and regional (HR = 1.39; 95% CI 1.30‐1.49) disease (Appendix Table S4). To better estimate the impact of TTC delays among different breast cancer subtypes, we performed a sensitivity analysis among patients with known breast tumor subtype (N = 10 340). The association between TTC delay and increased risk of death was evident among all BC subtypes (hormone receptor positive [HR = 1.46; 95% CI 1.15‐1.84], HER2‐positive [HR = 1.66; 95% CI 1.17‐2.38], and TNBC [HR = 2.18; 95% CI 1.63‐2.91]). Delays were also associated with worse BCSS among women with HER2‐positive disease (HR = 1.99; 95% CI 1.04‐3.79) and TNBC (HR = 2.15; 95% CI 1.38‐3.36) (Table 4).

**TABLE 3:** Multivariable Cox proportional hazards models with propensity score based to weights for overall survival and breast cancer‐specific survival according to time to chemotherapy (N = 28 968)^a^

	Overall survival			Breast cancer‐specific survival		
	Hazard ratio	95% CI	*P*	Hazard ratio	95% CI	*P*
Time from surgery to chemo						
0 to 30 d	1			1		
31 to 60 d	1.0	0.95 to 1.06	1.0	1.0	0.91 to 1.11	0.92
61 to 90 d	1.05	0.99 to 1.11	0.12	1.05	0.96 to 1.16	0.28
>90 d	1.25	1.18 to 1.32	<0.001	1.30	1.18 to 1.44	<0.001

Abbreviations: CI, confidence interval.

^a^ Models were additionally adjusted for region, year of diagnosis, age of diagnosis, race, marital status, Charlson comorbidity, tumor size, lymph node status, tumor grade, hormonal receptor status, surgery, radiation ≤1 y post diagnosis, state buy‐in, education and emergency room/hospitalization/complication.

**TABLE 4:** Multivariable Cox proportional hazards models with propensity score based to weights for overall survival and breast cancer‐specific survival according to time to chemotherapy and breast cancer subtype among patients of diagnosed 2010 to 2015 (N = 10 340)^a^

	Overall survival			Breast cancer‐specific survival		
	Hazard ratio	95% CI	*P*	Hazard ratio	95% CI	*P*
*Hormone receptor positive group (N = 5460)*						
TTC						
0 to 30 d	1			1		
31 to 60 d	1.02	0.78 to 1.32	.9	1.31	0.79 to 2.17	.30
61 to 90 d	1.23	0.96 to 1.57	.11	1.51	0.92 to 2.47	.11
>90 d	1.46	1.15 to 1.84	.002	1.56	0.97 to 2.51	.06
*HER2 positive group (N = 2843)*						
Time from surgery to chemo						
0 to 30 d	1			1		
31 to 60 d	1.35	0.96 to 1.9	.08	1.37	0.72 to 2.59	.34
61 to 90 d	1.59	1.15 to 2.2	.005	2.34	1.32 to 4.13	.003
>90 d	1.66	1.17 to 2.38	.005	1.99	1.04 to 3.79	.036
*Triple negative group (N = 2037)*						
Time from surgery to chemo						
0 to 30 d	1			1		
31 to 60 d	1.12	0.82 to 1.55	.48	1.15	0.70 to 1.88	.58
61 to 90 d	1.43	1.05 to 1.95	.025	1.39	0.86 to 2.24	.18
>90 d	2.18	1.63 to 2.91	<.001	2.15	1.38 to 3.36	<.001

Abbreviation: CI, confidence interval.

^a^ Models were additionally adjusted for age of diagnosis, Charlson comorbidity, and tumor grade.

## 4 DISCUSSION

Our study demonstrates in a large and representative cohort of BC patients that delaying TTC in older women is associated with statistically significant worse OS and BCSS. The association between TTC and survival was evident across all BC subtypes irrespective of extent of disease. Our findings are crucially important since women aged ≥65 comprised approximately 59% of all new BC cases in 2019.^18^ The benefit of adjuvant chemotherapy in early‐stage BC patients is well‐established. A retrospective study of women age >65 with hormone‐receptor negative early stage BC showed a 15% mortality reduction associated with adjuvant chemotherapy.^19^ However, older women are usually underrepresented in clinical trials and less likely to receive guideline‐concordant treatment.

Suboptimal treatment among older patients has important implications in cancer outcomes. BC mortality decreased from 1975‐2000^20^; however, an analysis of National Vital Statistics Reports from 1990 to 2007 and SEER data from 1980 to 1997 demonstrated that BC outcomes have improved more significantly in women <75 compared to their older counterparts.^21^ While BC death rates in younger women were stable from 1980 to 1989, they increased in older women.^21^ BC death rates began to decrease across all age groups in 1990, but the decrease among older women was <50% of the decrease among younger women.^21^

While we observed adverse outcomes among all BC subtypes, our study suggests that the impact of TTC on survival is greater among women with hormone receptor‐negative and HER2‐positive disease, compared those with hormone receptor‐positive disease. TNBC and HER2‐positive BC have a more aggressive biology.^22^, ^23^ Furthermore, some studies suggest that the absolute benefit of adjuvant chemotherapy is greater among patients with highly proliferative tumors such as HER2‐positive and TNBC for whom chemotherapy can be highly effective.^24^ Given that adjuvant endocrine therapy greatly reduces the risk of recurrence in hormone receptor‐positive BC,^25^, ^26^, ^27^ the survival impact of TTC in these patients may be also due to delayed initiation of endocrine therapy. We observed that the relationship between TTC and mortality varies by tumor subtype. While a TTC > 90 days after surgery is associated with worse OS among all tumor subtypes, we observed an increased risk of death among patients with HER2‐positive (HR = 1.59, 95% CI 1.15‐2.2) and triple negative (HR = 1.43, 95% CI 1.05‐1.95) tumors even when TTC was >60 days. These findings suggest that among patients with highly proliferative tumors, the optimal time window to start adjuvant chemotherapy is narrower. However, our study still demonstrates a time‐dependent survival benefit even among patients that received chemotherapy >90 days after surgery.

Our group reported an association between TTC and survival in a prior study using data from the CCR^4^ and data of patients treated at MD Anderson Cancer Center (MDACC),^5^ though these studies did not focus on older women. In the CCR cohort, 29.6% of the patients were age >60, while the median age of the MDACC cohort was 50 years. Because the data from our current study comes from Medicare‐linked databases, all patients in the cohort are over age 65 years. However, our study shows that even among a cohort age >65, increasing age was associated with increased TTC. Our current study is unique since it focused on elderly breast cancer patients, but combined with our previous work, supports the body of evidence of the negative survival outcomes associated with delaying TTC.

In this study, we were able to identify demographic and clinical factors associated with delayed TTC. Patients with hormone receptor‐negative tumors were less likely to experience TTC delays compared with hormone receptor‐positive patients, it is possible that there was likely a greater sense of urgency among providers to initiate chemotherapy in patients with these tumor types given their more aggressive behavior. We also observed that patients whose tumors were sent for Oncotype DX testing and who received adjuvant radiation before chemotherapy were more likely to experience delays. Given the worse survival outcomes associated with adjuvant chemotherapy delays, providers should engage proactively other members of their patients’ multidisciplinary care teams to coordinate diagnostics and treatment modalities efficiently. It is possible that delays with increasing age are associated with concerns about older patients’ ability to tolerate chemotherapy without significant adverse events.^28^, ^29^ Not surprisingly, we observed that delays were more likely among women with more comorbidities as well as ER visits, hospitalizations, and surgical complications. Our study shows that race/ethnicity are associated with an increased risk of TTC delays. Racial disparities in BC have been well‐described and may be related to differences in access to care.^4^, ^6^ A 2013 study using SEER‐Medicare data found the mean time from diagnosis to treatment for African‐American women was 29.3 days compared to 22.5 days for white women.^30^ A 2004 case‐control study of women diagnosed with breast cancer in Atlanta, GA reported delays in chemotherapy initiation >90 days from diagnosis in 22.4% of African American women versus 14.3% in white women, despite adjusting for SES and other factors.^31^

This is the largest study evaluating the impact of TTC among older BC patients. We provide clear evidence that timely chemotherapy administration is crucial and delays are associated with worse OS and BCSS regardless of BC subtype. Our observations are strengthened by the large number of patients, long follow‐up time, and use of the IPTW method to adjust for potential selection bias. Our study, however, is limited by its retrospective nature. Despite the methodological limitations inherent to claims‐based research, observational studies are of clinical value and they provide real‐life data that is generalizable allowing identification of patters of care and areas where cancer care delivery can be improved. Given the study design limitations and ethical considerations associated to the evaluation of treatment delays, observational studies from real word data might be the only way to examine the impact of delayed chemotherapy on survival.

Our study is limited by the variables available in the databases. We could not account for delays in diagnosis, delays in initiating adjuvant endocrine therapy or distance from patient's home to the nearest hospital. Additionally, BCSS information could be inaccurate due to misclassification of cause of death in the SEER‐Medicare and TCR‐Medicare databases. Oncotype DX results for those who had testing was not available. This information would have allowed us to stratify further hormone receptor‐positive and HER2‐negative patients to determine if the survival effect of TTC was more pronounced among patients with high recurrence scores. Furthermore, it is possible that longer follow‐up would reveal that TTC delays has a more pronounced survival impact in patients with hormone receptor‐positive BC where recurrence risk tends follow a longer course compared to HER2‐positive BC and TNBC.^32^

Another limitation is the exclusion of a significant proportion of patients because they were enrolled in HMO. Previous studies have shown a correlation between insurance type and TTC, and it is not something we were able to fully assess. However, we believe that our study is still fairly representation of the older adult population given the inclusion of patients enrolled in Medicare, which provides medical care for the vast majority of older Americans.^33^ Lastly, our study lacks information on patients’ preferences and decision‐making regarding TTC. Despite these limitations, our study provides an in‐depth evaluation of the impact of treatment delays in a vulnerable population.

In conclusion, our study adds to the body of evidence and confirms that delayed TTC is associated with worse survival among BC patients >65. Decreased OS was also seen with TTC > 60 days in patients with regional disease and those with HER2‐positive or TNBC. This data should encourage providers to take appropriate steps to ensure timely initiation of chemotherapy. In addition to medical factors and age, we found that socioeconomic factors such as race and marital status were associated with delays in care. Future studies should attempt to determine the reasons such disparities exist in order to develop effective strategies to decrease gaps in cancer care delivery.

## CONFLICT OF INTEREST

None.

## AUTHOR CONTRIBUTIONS

Demetria Smith‐Graziani contributed to investigation, visualization, writing—original draft, writing—review and editing. Xiudong Lei contributed to conceptualization, data curation and formal analysis, investigation, writing—review and editing. Sharon Giordano contributed to conceptualization, funding acquisition, writing—review and editing. Hui Zhao contributed to data curation and formal analysis, investigation, methodology, writing—review and editing. Meghan Karuturi contributed to writing—review and editing. Mariana Chavez‐MacGregor contributed to conceptualization, data curation and formal analysis, funding acquisition, methodology, supervision, writing—original draft, writing—review and editing.

## Supporting information

Supplementary Material

## Data Availability

The data that support the findings of this study are openly available in the SEER‐Medicare database at https://healthcaredelivery.cancer.gov/seermedicare.
