# Supplementary material for: Delayed initiation of adjuvant chemotherapy in older women with breast cancer
Source: Cancer Med. 2020 Aug 7;9(19):6961–71. doi: 10.1002/cam4.3363 (PMC7541132; doi:10.1002/cam4.3363)
Supplement: Supplementary file 1 — Supplementary Material [file CAM4-9-6961-s001.docx]

**Appendix Table A1.** SEER/TCR-Medicare cohort

|  | SEER/TCR-Medicare 2001-2015 | Total |  |
| --- | --- | --- | --- |
| **Step** | **Cohort Selection Criteria** | Met Criteria | Excluded |
| 1 | Included patients with breast cancer with Sitewho1 = 26000. | 1,009,841 |  |
| 2 | Included year of diagnosis 2001 - 2015 but not diagnosed at autopsy or death certificate only. | 711,243 | 298,598 |
| 3 | Included female. | 706,016 | 5,227 |
| 4 | Included patients who were age 66 years and older at diagnosis. | 388,038 | 317,978 |
| 5 | Included localized or regional stages. | 287,241 | 100,797 |
| 6 | Included patients whose first Primary Cancer was breast cancer and no 2nd within 12 months. | 245,311 | 41,930 |
| 7 | Included patients with histology confirmation. | 243,925 | 1,386 |
| 8 | Included patients who enrolled in Medicare Part A & B +/- 12 months or until death. | 227,751 | 16,174 |
| 9 | Excluded patients who enrolled in Health Maintenance Organization +/-12 months or until death. | 156,410 | 71,341 |
| 10 | Included patients who received lumpectomy, mastectomy between 90 days prior to diagnosis and 1 year after diagnosis. | 127,528 | 28,882 |
| 11 | Included patients who received chemotherapy after surgery (including day 0) and within 9 months of surgery. | 31,499 | 96,029 |
| 12 | Excluded patients who received neoadjuvant chemotherapy. | 28,968 | 2,531 |
| TOTAL NUMBER OF PATIENTS IN BREAST CANCER COHORT | | 28,968 |  |

**Appendix Table A2.** Codes used to identify treatment

| **Simplified Group Description** | **Revenue Center Codes** | **Diagnosis Codes** | **Procedure Codes** | **HCPCS Codes** |
| --- | --- | --- | --- | --- |
| Lumpectomy / Excisional biopsy |  |  | ICD-9 codes:  85.12, 85.2, 85.20, 85.21, 85.22, 85.24, 85.25  ICD-10 codes:  0H9T0ZX, 0H9U0ZX,0H9V0ZX,0H9W0ZX,  0H9X0ZX,0HBT0ZX,0HBU0ZX,0HBV0ZX,  0HBW0ZX,0HBX0ZX,0HBY0ZX,0H5T0ZZ,  0H5T3ZZ,0H5T7ZZ,0H5T8ZZ,0H5TXZZ,  0H5U0ZZ,0H5U3ZZ,0H5U7ZZ,0H5U8ZZ,  0H5UXZZ,0H5V0ZZ,0H5V3ZZ,0H5V7ZZ,  0H5V8ZZ,0H5VXZZ,0HBT0ZZ,0HBT3ZZ,  0HBT7ZZ,0HBT8ZZ,0HBTXZZ,0HBU0ZZ,  0HBU3ZZ,0HBU7ZZ,0HBU8ZZ,0HBUXZZ,  0HBV0ZZ,0HBV3ZZ,0HBV7ZZ,0HBV8ZZ,  0HBVXZZ,0HBY0ZZ,0HBY3ZZ,0HBY7ZZ,  0HBY8ZZ,0HBYXZZ,0HTY0ZZ,0H5W0ZZ,  0H5W3ZZ,0H5W7ZZ,0H5W8ZZ,0H5WXZZ,  0H5X0ZZ,0H5X3ZZ,0H5X7ZZ,0H5X8ZZ,  0H5XXZZ,0HBW0ZZ,0HBW3ZZ,0HBW7ZZ,  0HBW8ZZ,0HBWXZZ,0HBX0ZZ,0HBX3ZZ,  0HBX7ZZ,0HBX8ZZ,0HBXXZZ,0HTWXZZ,  0HTXXZZ | 19120, 19125, 19126, 19160, 19301 |
| Lumpectomy and lymph node combined procedures |  |  |  | 19162, 19302 |
| Mastectomy |  |  | ICD-9 codes:  85.40-85.42, 85.34, 85.36  ICD-10 codes:  0HBT0ZZ,0HBT3ZZ,0HBU0ZZ,0HBU3ZZ,  0HBT0ZZ,0HBT3ZZ,0HBV0ZZ,0HBV3ZZ,  0HTT0ZZ,0HTU0ZZ,0HTV0ZZ | 19180, 19303, 19182, 19304 |
| Mastectomy and lymph node combined procedures |  |  | ICD-9 codes:  85.43-85.48  ICD-10 codes:  07T50ZZ,0HTT0ZZ,07T60ZZ,0HTU0ZZ,  0HTV0ZZ,07T70ZZ,07T80ZZ,0HTT0ZZ,  07T60ZZ,07T70ZZ,07T90ZZ,0HTU0ZZ | 19240, 19307, 19220, 19306, 19200, 19305 |
| Lymph node excision |  |  | ICD-9 codes:  40.11, 40.22, 40.23, 40.29, 40.3, 40.5,  40.50, 40.51  ICD-10 codes:  07B80ZZ,07B83ZZ,07B84ZZ,07B90ZZ,  07B93ZZ,07B94ZZ,07B50ZZ,07B53ZZ,  07B54ZZ,07B60ZZ,07B63ZZ,07B64ZZ,  07T54ZZ,07T60ZZ,07T64ZZ | 38500, 38525, 38530, 38740, 38745 |
| General reconstruction |  |  | ICD-9 codes:  85.50, 85.7, 85.70, 85.8  ICD-10 codes:  0H0T07Z,0H0T0JZ,0H0T0KZ,0H0T37Z,  0H0T3JZ,0H0T3KZ,0H0TX7Z,0H0TXJZ,  0H0TXKZ,0H0U07Z,0H0U0JZ,0H0U0KZ,  0H0U37Z,0H0U3JZ,0H0U3KZ,0H0UX7Z,  0H0UXJZ,0H0UXKZ,0H0V07Z,0H0V0JZ,  0H0V0KZ,0H0V37Z,0H0V3JZ,0H0V3KZ,  0H0VX7Z,0H0VXJZ,0H0VXKZ, 0HRT07Z,  0HRT0JZ,0HRT0KZ,0HRU07Z, 0HRU0JZ,  0HRU0KZ | 19324, 19366 |
| Implant reconstruction |  |  | ICD-9 codes:  85.33, 85.35, 85.53, 85.54, 85.95, 85.99  ICD-10 codes:  0H0T0JZ,0H0T3JZ,0H0U0JZ,0H0U03JZ,  0HRT0JZ,0HRT3JZ,0HRU0JZ,0HRU3JZ,  0HRV0JZ,0HRV3JZ,0H0V0JZ,0H0V3JZ,  0H0T0KZ,0H0T3KZ,0H0U0KZ,0H0U3KZ,  0HRT0KZ,0HRT3KZ,0HRU0KZ,0HRU3KZ,  0HUT0JZ,0HUT0KZ,0HUT3JZ,0HUT3KZ,  0HUU0JZ,0HUU0KZ,0HUU3JZ,0HUU3KZ,  0H0V07Z,0H0V0KZ,0H0V37Z,0H0V3KZ,  0HUV0JZ,0HUV3JZ,0HHT0NZ,0HHT3NZ,  0HHT7NZ,0HHT8NZ,0HHU0NZ,0HHU3NZ,  0HHU7NZ,0HHU8NZ,0HHV0NZ,0HHV3NZ,  0HHV7NZ,0HHV8NZ,0HHW0NZ,0HHW3NZ,  0HHW7NZ,0HHW8NZ,0HHX0NZ,0HHX3NZ,  0HHX7NZ,0HHX8NZ | 19325, 19340, 19357 |
| Autogenous tissue flap reconstruction |  |  | ICD-9 codes:  85.71-85.76, 85.79, 85.84, 85.85  ICD-10 codes:  0HRT075,0HRU075,0HRV075,0KXK0Z6,  0KXK4Z6,0KXL0Z6,0KXL4Z6,0HRT076,  0HRU076,0HRV076,0HRT077,0HRU077,  0HRV077,0HRT078,0HRU078,0HRV078,  0HRT079,0HRU079,0HRV079,0HRT07Z,  0HRT0JZ,0HRT0KZ,0HRU07Z,0HRU0JZ,  0HRU0KZ,0HX5XZZ,0KXH0ZZ,0KXH4ZZ,  0KXJ0ZZ,0KXJ4ZZ | 19364, 19361, 19362, 19367, 19368, 19369, 69990, 09920 |
| Radiation (actual delivery) External beam radiation therapy |  |  |  | 77401-77416, 77418, 77422, 77423, 77522-77525, 0073T, G0174 |
| Radiation (actual delivery) Brachytherapy |  |  | ICD-9 codes: 92.27 | 77761-77763, 77767-77768, 77770-77772, 77776-77778, 77781-77790, 77799 |
| Chemotherapy | 0331, 0332, 0335 | ICD-9 codes:  V58.1, V66.2, V67.2 | ICD codes: 99.25 | 96400 – 96549, J9000 - J9999 (excluding J9003, J9165, J9175, J9202, J9209, J9240, J9395),  J8520, J8521, J8530, J8540, J8560, J8597, J8610, J8999  Q0083 – Q0085 |
| Lymph node positive |  | ICD-9 codes: 196.3, 195.1 |  |  |
| Trastuzumab |  |  |  | J9355, C9292, C9131 |
| Oncotype DX Test |  |  |  | 84999, S3845 |
| Surgical complication |  | ICD-9 codes: 459.0, 682.2, 611.0, 998.32, 998.13, 996.69 |  | 10180,11971,11042-11047,97597,97598 |

ICD-9, International Classification of Diseases, 9th revision

ICD-10, International Classification of Diseases, 10th revision

HCPCS, Healthcare Common Procedure Coding System

**Appendix Table A3.** Multivariable Cox proportional hazards models with propensity score based to weights for overall survival and breast cancer-specific survival according to time to chemotherapy (N=28,968)*

|  | **Overall Survival** | | | **Breast Cancer-Specific Survival** | | |
| --- | --- | --- | --- | --- | --- | --- |
|  | **Hazard Ratio** | **95% CI** | **P** | **Hazard Ratio** | **95% CI** | **P** |
| Time from surgery to chemo |  |  |  |  |  |  |
| 0 to 90 days | 1 |  |  | 1 |  |  |
| 91 to 120 days | 1.13 | 1.07 to 1.20 | <0.001 | 1.04 | 0.94 to 1.15 | 0.42 |
| 121 to 180 days | 1.27 | 1.18 to 1.36 | <0.001 | 1.59 | 1.42 to 1.77 | <0.001 |
| >180 days | 1.56 | 1.42 to 1.71 | <0.001 | 1.80 | 1.53 to 2.10 | <0.001 |

*Models were additionally adjusted for region, year of diagnosis, age of diagnosis, race, marital status, Charlson comorbidity, tumor size, lymph node, tumor grade, hormonal receptor status, surgery, emergency room / hospitalization / complication, radiation <1 year post diagnosis, state buy to in, and education.

Abbreviations: CI, confidence interval.

**Appendix Table A4.** Multivariable Cox proportional hazards model with propensity score based-weights for overall survival and breast cancer-specific survival according to time to chemotherapy according to receptor status and stage*

|  | **Overall Survival** | | | **Breast Cancer-Specific Survival** | | |
| --- | --- | --- | --- | --- | --- | --- |
|  | **Hazard Ratio** | **95% CI** | **P** | **Hazard Ratio** | **95% CI** | **P** |
| **Hormonal receptor positive group**  **(N=17,583)** |  |  |  |  |  |  |
| Time from surgery to chemo |  |  |  |  |  |  |
| 0-30 days | 1 |  |  | 1 |  |  |
| 31-60 days | 1.04 | 0.97 to 1.13 | 0.26 | 1.07 | 0.93 to 1.22 | .52 |
| 61-90 days | **1.11** | **1.02 to 1.19** | **0.01** | **1.15** | **1.01 to 1.32** | **.042** |
| >90 days | **1.44** | **1.34 to 1.56** | **<0.001** | **1.49** | **1.30 to 1.71** | **<.001** |
| **Hormonal receptor negative group**  **(N=7,195)** |  |  |  |  |  |  |
| Time from surgery to chemo |  |  |  |  |  |  |
| 0-30 days | 1 |  |  | 1 |  |  |
| 31-60 days | 1.04 | 0.93 to 1.15 | 0.48 | 0.99 | 0.84 to 1.16 | .88 |
| 61-90 days | 1.10 | 0.99 to 1.22 | 0.08 | 1.03 | 0.88 to 1.21 | .73 |
| >90 days | **1.17** | **1.04 to 1.3** | **0.006** | **1.20** | **1.02 to 1.42** | **.031** |
| **Localized stage group**  **(N=13,370)** |  |  |  |  |  |  |
| Time from surgery to chemo |  |  |  |  |  |  |
| 0-30 days | 1 |  |  | 1 |  |  |
| 31-60 days | 0.97 | 0.88 to 1.07 | 0.57 | 1.11 | 0.89 to 1.37 | .35 |
| 61-90 days | 1.02 | 0.92 to 1.13 | 0.76 | 0.99 | 0.79 to 1.23 | .92 |
| >90 days | **1.19** | **1.07 to 1.32** | **<0.001** | **1.46** | **1.18 to 1.81** | **<.001** |
| **Regional stage group**  **(N=15,598)** |  |  |  |  |  |  |
| Time from surgery to chemo |  |  |  |  |  |  |
| 0-30 days | 1 |  |  | 1 |  |  |
| 31-60 days | 1.04 | 0.97 to 1.11 | 0.29 | 0.99 | 0.89 to 1.10 | .85 |
| 61-90 days | **1.13** | **1.06 to 1.21** | **<0.001** | 1.13 | 1.02 to 1.25 | .023 |
| >90 days | **1.39** | **1.30 to 1.49** | **<0.001** | **1.39** | **1.25 to 1.54** | **<.001** |

*Models were adjusted for region, year of diagnosis, age of diagnosis, race, marital status, Charlson comorbidity, stage (if appropriate), grade, hormonal receptor status (if appropriate), surgery, radiation in 1 year post diagnosis, state buy-in, and education.

Abbreviations: CI, confidence interval.
